# Controlled Morphological Bending of 3D-FEBID Structures via Electron Beam Curing

**DOI:** 10.3390/nano12234246

**Published:** 2022-11-29

**Authors:** Anna Weitzer, Robert Winkler, David Kuhness, Gerald Kothleitner, Harald Plank

**Affiliations:** 1 Institute of Electron Microscopy and Nanoanalysis, Graz University of Technology, 8010 Graz, Austria; 2 Christian Doppler Laboratory for Direct-Write Fabrication of 3D Nano-Probes, Institute of Electron Microscopy and Nanoanalysis, Graz University of Technology, 8010 Graz, Austria; 3 Graz Centre for Electron Microscopy, Steyrergasse 17, 8010 Graz, Austria

**Keywords:** additive manufacturing, direct-write nano-fabrication, focused electron beam induced deposition, 3D nanoprinting, metal nanostructures, electron beam curing, electron trajectory simulations, post processing

## Abstract

Focused electron beam induced deposition (FEBID) is one of the few additive, direct-write manufacturing techniques capable of depositing complex 3D nanostructures. In this work, we explore post-growth electron beam curing (EBC) of such platinum-based FEBID deposits, where free-standing, sheet-like elements were deformed in a targeted manner by local irradiation without precursor gas present. This process diminishes the volumes of exposed regions and alters nano-grain sizes, which was comprehensively characterized by SEM, TEM and AFM and complemented by Monte Carlo simulations. For obtaining controlled and reproducible conditions for smooth, stable morphological bending, a wide range of parameters were varied, which will here be presented as a first step towards using local EBC as a tool to realize even more complex nano-architectures, beyond current 3D-FEBID capabilities, such as overhanging structures. We thereby open up a new prospect for future applications in research and development that could even be further developed towards functional imprinting.

## 1. Introduction

When building complex structures, there are a number of additive manufacturing methods available for generating three-dimensional elements on the macro- and microscale. However, with decreasing structure sizes, only a handful of possible techniques remain [1,2,3], such as laser-assisted electrophoretic deposition [4], electro-chemical printing [5], or two-/multiphoton polymerization [6], to name a few [7,8]. Another nanoscale technology, capable of depositing advanced objects with feature sizes down to 20 nm under optimized conditions [9], is focused electron beam-induced deposition (FEBID) [10]. This increasingly relevant method is capable of creating sophisticated three-dimensional architectures with exceedingly high resolution in a single-step procedure and is thereby a powerful tool for controlled high-precision fabrication of free-standing 3D nanostructures [11,12]. Other advantages of this technique include low demands on substrate morphologies and materials, object scalability, and a constantly growing number of available precursors [13]. The working principle of FEBID is based on the interplay between a precisely controllable, focused electron beam and surface-adsorbed precursor molecules that were injected into the microscope vacuum chamber in a gaseous state. Primary, scattered and especially secondary electrons can trigger dissociation processes of precursor molecules [14,15], which immobilizes the non-volatile core components of the molecules, while the volatile parts are diverted by the vacuum pump system. Consequently, the former remain on the surface and form the intended deposit [16]. The navigation of the electron beam in the XY-plane and the precise adjustment of the dwell times (deposition times in each individual position) ultimately generate the deposit morphology [17]. Especially 3D-FEBID, where true three-dimensional architectures are built via slow lateral movements, is a very powerful tool that enables novel application concepts for plasmonics [12], nanomagnetics [18,19,20] and 3D-nanoprobe fabrication for scanning probe microscopy [21,22], among others. The technique that was mainly used to build mesh-like structures in the past [23,24], was recently expanded to closed or sheet-like objects, which strongly improved its options in terms of design flexibility [25,26,27,28]. This transition, however, entailed new challenges in terms of growth stability and spatially varying growth rates depending on the object dimensions as well as the position of the deposition point within said structure. Since the most commonly used precursor (MeCpPt^(IV)^Me_3_) notoriously exhibits rather high carbon contents, due to incomplete dissociation processes, the deposited structures hold low metal contents around 15 at.% [29,30]. This has a tremendous impact on the temperature conditions during the growth process, as the energy brought into the system by the electron beam can only be dissipated towards the substrate through the carbon-dominated material, which is a poor thermal conductor [28,31]. This geometry-dependent electron beam heating [32,33] leads in turn to an increased desorption of precursor molecules, which slows down the growth rate [34], leading to non-uniform conditions for deposition, especially for narrow but tall 3D nanostructures. The deviations of the deposited object shapes from originally intended geometries can be compensated to some extent by adjusting the individual dwell times accordingly [27,28]. For receiving higher electrical and thermal conductivities [35] and/or changing the mechanical properties of the material [36], different post-growth approaches have been introduced, that enable precise modification of the nano-granularity [37,38] or even the entire removal of carbon [29,30,39]. This, however, can disrupt the original morphology of the deposited objects. The here relevant post-processing technique is electron beam curing (EBC) [35,40], which exposes the grown structures to an electron beam once again, this time, however, without precursor gas present. Effectively, this method proceeds with the dissociation process of incompletely fragmented but incorporated molecules [13], most likely assisted by residual water molecules in the vacuum as reactants and thermal effects due to electron beam heating. This leads to slightly growing nano-grains, a modification of the carbon matrix and small volume losses due to the formation of volatile fragments upon dissociation [36,38]. As unintentional deformations of 3D-FEBID structures, especially pillars, were also observed after imaging and even as a co-process during fabrication in the past [41], different theories were developed, ascribing the effect to various causes such as mechanical stress, local charging, temperature effects, or chemical modifications [41,42]. Following the latter path, the idea of using localized electron beam curing for the targeted deformation of sheet-like 3D architectures arose. Based on that motivation, we tested various parameters and curing pattern dimensions for controlled geometric alterations, such as the bending of vertical walls or other deposited base structures via rectangular curing strips, among others. SEM, TEM and AFM investigations were performed to uncover the underlying EBC processes, verifying the assumption that the inner structure, in terms of grain size, the density of the material and its volume are changed at cured regions. We were able to receive systematic, controlled deformations depending on the primary electron energies, the irradiation dose and the beam incidence angles during curing and substantiate our results with Monte Carlo simulations. This way we even achieved the fabrication of overhanging structures, a geometry that cannot be directly deposited with 3D-FEBID. With these first basic investigations, we open up the range of possible future applications for this post-growth processing technique, potentially even into the direction of functional imprinting or changed oscillation modes, as electron beam cured areas exhibit slightly changed properties compared to as-deposited materials, which could be utilized for various applications in research and development.

## 2. Results and Discussion

**Figure 1:**
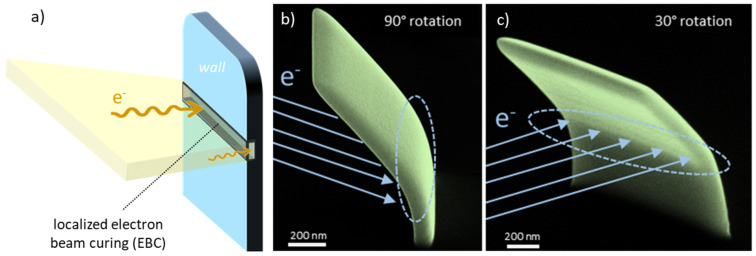
Illustration of the wall bending process via localized electron beam curing (EBC). (**a**) 3D schematic of the original, straight wall structure and the EBC patterning geometry. Such treatments lead to a controlled bending, as shown by SEM images of a bent wall element under 52° tilt at rotation angles of 90° (**b**) and 30° (**c**), delivering the proof-of-principle.

When closed or mesh-like FEBID structures are locally irradiated by electrons, targeted bending via EBC becomes possible, presumably due to structural and volumetric changes caused by reordering processes. To test this behaviour, we deposited vertical wall elements via FEBID and irradiated them again via horizontal patterning strips, as shown by the 3D scheme in Figure 1a. A representative result is shown in Figure 1b,c, which provides the proof-of-principle for the here-targeted post-growth deformation process via EBC.

**Figure 2:**
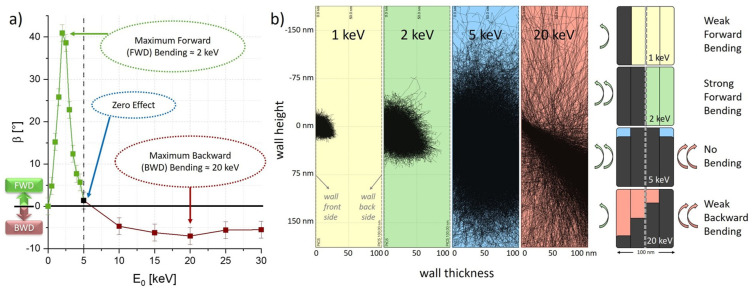
Implications of primary electron energies on EBC bending. (**a**) Experimentally bent angles β depending on $\;E_{0}$, with forward and backward bending illustrated in green and red, respectively. (**b**) Monte Carlo electron trajectory simulations (left) for selected primary electron energies applied to 100 nm thick $PtC_{5}$ elements. The schemes on the right illustrate the EBC regions in dark grey, together with unaffected parts using the same colour code. The estimated extent of EBC is shown by the bar heights, which becomes particularly relevant for the 20 keV situation, where the strong spreading becomes evident.

**Primary Electron Energies**—To identify ideal parameters for reproducible and effective bending via EBC, we first focus on implications of varying primary electron energies $E_{0}$. As a test design, we chose vertical FEBID walls with widths W=1000 nm, heights H=2000 nm and thicknesses T=100 nm, that were deposited at 5 keV and 40 pA by using our recently introduced compensation tool [28]. The bending process was conducted at beam currents of ≈40 pA via patterning arrays of H×W=200 nm×1100 nm with constant point pitches of PoP=25 nm, dwell times of DT=1 ms, 1000 passes, and an electron beam incidence angle of α=52°, thereby maintaining the same overall doses of 7.4 C/cm². The resulting bending angles β are depicted in Figure 2a, showing not only a strong $E_{0}$ dependency for the formerly observed forward bending process, but also the possibility of backward bending for higher primary electron energies. As evident, the strongest effect for this specific wall thickness was achieved for $E_{0}\approx 2\;\mathrm{keV}$ and $E_{0}\approx \;20\;\mathrm{keV}$ for forward (green) and backward bending (red), respectively. The reason for this behaviour lies in the spatial distribution of energy deposition according to penetration depths, which leads to structural and volumetric changes not only in the outmost layer, but inside the wall as well. In other words, EBC-affected regions are proposed to contract, which leads to a stress–strain-induced bending effect if these regions are asymmetrically distributed across the wall thickness, as it seems to be ideal for $E_{0}\approx 2\;\mathrm{keV}$. For decreasing energies, the affected volumes get too small to induce strong bending, above 2 keV, the bending effect also decreases, as penetration depths travel deeper inside the wall, therefore generating a more symmetric stress–strain situation. At 5 keV, bending stops entirely, which suggests that EBC is now fully spread across the wall thickness. For even higher energies, EBC travels more and more to the backside, which again induces an asymmetric situation, this time, however, leading to backward deformation. The reason why backward bending is not as efficient is attributed to (1) an increasing number of transmitted electrons, which reduces the EBC effect, and (2) the fact that the scattered electrons spread out over the entire wall thickness, with just a bias towards the backside but with reduced defined asymmetry.

Complementary Monte Carlo simulations with Casino [43] support the aforementioned assumptions. Figure 2b compares the electron interaction volumes for four selected primary electron energies, for wall thicknesses of 100 nm under a 52° tilt angle, to illustrate spatial differences. For $E_{0}=1\;\mathrm{keV}$ (yellow) the electron interaction volume only covers the foremost wall layer, causing weak forward bending, which is also depicted in the related illustration on the right with irradiated wall fractions schematically shown in dark grey. For $E_{0}=2\;\mathrm{keV}$ (green) strong forward bending occurs, due to the fact that the extent of the interaction volume covers roughly 50% of the wall thickness (as shown in the right-hand side illustration). Primary electron energies of 5 keV (blue) lead to interaction volumes that are symmetrically expanded throughout the entire wall thickness. As this is a geometrically balanced process, hardly any deformations are visible in experiments. For 20 keV (red) the electron interaction volume is altered to a cone-like shape as the majority of primary electrons pass through the structure and leave on the backside. Even though there is EBC across the entire wall due to stronger spreading of scattering, there is more energy deposition towards the backside of the structure, leading to the aforementioned backward bending effect. The less sharp asymmetry is finally responsible for the weaker bending effect in the backwards direction.

**Figure 3:**
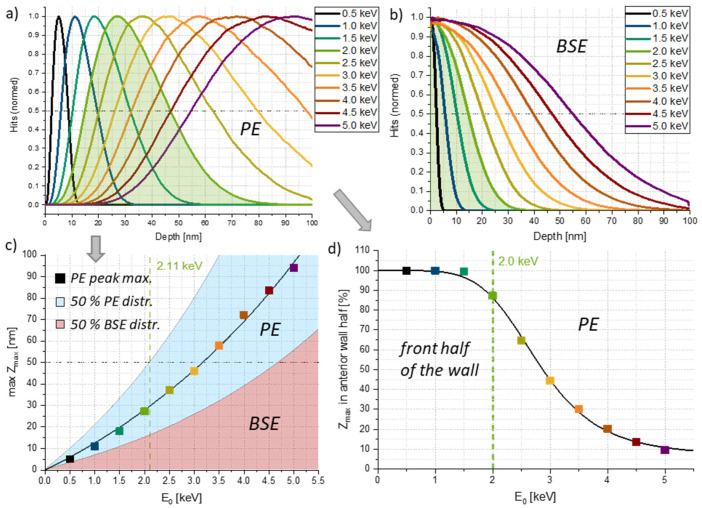
Monte Carlo simulations for different primary electron energies. (**a**) Maximum penetration depths of primary electrons (PE) with the experimentally found ideal value of 2 keV filled green. (**b**) gives the same information for backscattered electrons (BSE), (**c**) summarizes the peak positions from (**a**) and expands them by the full-width-half-maximum values for both PE (blue shaded) and BSE penetration depths (red shaded). (**d**) shows the energy-dependent percentage of primary electrons found in the anterior half of the wall.

The analysis of a series of simulations for varying $E_{0}$ reinforced the results from our experiments, as summarized in Figure 3. The data compilation shows the varying maximum penetration depths $Z_{max}$, with the experimentally found ideal energy of 2 keV filled green for (a) primary electrons (PE) and (b) backscattered electrons (BSE), which are also generated during EBC. A closer look into (a) suggests, that an energy of 1.5 keV might be even more suited to achieve a highly symmetric 50:50 cured-uncured situation. However, further effects have to be considered. First, the distribution around the penetration depth maxima has to be taken into account, as shown in (c) for PE by the full-width-half-maximum values as blue shaded part around their peak maxima. As evident, 2 keV seems to cover the front half to a far extent, although not entirely. That leads to the second aspect, which is the BSE contributions (full-width-half-maximum values as red shaded area). From Figure 3b, it becomes evident, that for energies >2 keV electrons start to reach into the second half of the wall thickness, which is not ideal for the bending mechanism. Particularly interesting is the contribution of 5 keV PEs and BSEs, where the former are mainly found in the back part, while the latter cover the front part, leading to full EBC across the entire wall thickness, without any bending. For a clearer picture in agreement with our model, Figure 3d depicts the percentage of primary electrons with maximum penetration depths on the anterior 50% of the wall. As evident, for energies up to 1.5 keV, practically all electrons remain within one half. For 2 keV, which was found as an ideal value during experiments (Figure 2), a decay to about 90% is found, which will be discussed again later, once the volume loss is quantified. Furthermore, we expect this value to increase when including the impact of backscattered electrons. For higher energies, the percentage is quickly decreasing in agreement with experimental findings.

Concerning the mechanism behind this deformation process, we briefly want to mention possible temperature impacts during EBC. We acknowledge that increased temperatures might trigger additional effects; however, for the given settings, variations of only a few degrees Celsius are expected, according to previous studies [9,32]. We therefore assume that temperature implications are not the predominant factor, the bending mechanism suggested here is rather related to volume loss and grain growth effects, which will be investigated in more detail further down.

**Figure 4:**
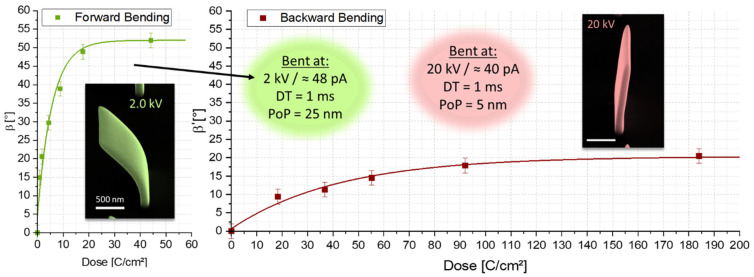
Bending angles depending on overall irradiation dose. Forward (green, left) and backward bending angles (red, right)  β and β’  respectively, with exemplary SEM images as insets, both show saturation limits.

**EBC Dose Variation**—After identifying the ideal $E_{0}$ ranges, dose variation experiments were conducted, as summarized in Figure 4. Both, forward (2 keV) as well as backward bending (20 keV), showed exponential saturation trends for increasing doses, which indicates the EBC limits. Hereby, it is noteworthy that low doses are already sufficient for targeted deformation in the forward direction, whereas backward bending only works to a certain extent and needs comparatively long irradiation times and doses. We ascribe this limit to the shape of the electron interaction volumes (compare Figure 2b), where, for forward bending, curing almost exclusively happens in the front part, whereas for backward bending there is EBC throughout the whole thickness of the walls but with a bias towards the backside, preventing a pure one-sided curing and thereby dampening the maximum possible deformation imbalance within the structures. Also noteworthy is the physical bending limit for forward bending of the structures due to the geometric positioning of the electron beam, which here comes from a tilt angle of 52°. Naturally, backward bending is not limited by geometric conditions as the deformation direction points away from the incoming beam.

**Figure 5:**
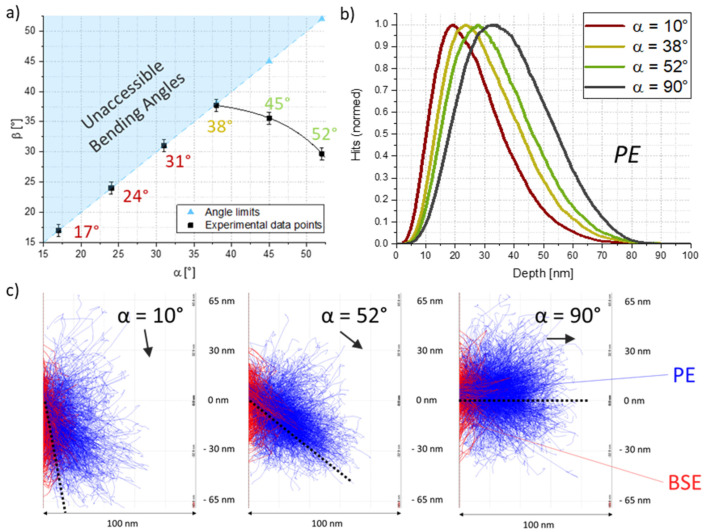
The effects of varying beam incidence angles at 2 keV and constant doses. (**a**) shows experimentally achieved bending angles β vs. beam incidence angles α together with the intrinsic, geometrical bending limits (blue). (**b**) Distributions of maximum penetration depths for different beam incidence angles, derived from Monte Carlo simulations, where increasing depths for higher angles are evident. (**c**) Electron trajectory simulations for 3 different beam incidence angles α separated in primary electrons (PE, blue) and backscattered electrons (BSE, red).

**Beam Incidence Angles**—To explore possible bending implications by varying beam angles α, we have conducted a dedicated study. As the shape of the electron interaction volume and the maximum penetration depths change with the angle in which the electrons intrude the wall surface, significant differences can be found in both, experiments and simulations. Theoretically favourable would be an invasion that is rather flat regarding the structure surface, as in this case the most energy could be introduced in the foremost wall area. In practice, the aforementioned geometrical bending limits have to be considered. Figure 5a shows experimentally found bending angles of single walls, that have been irradiated at 2 keV with a dose of 6.6 C/cm² under different beam incidence angles (black) together with the geometrical limits (blue). As evident, the advantage of more effective bending for lower α-values could only be accessed between 38° and 52°, where for lower α-values increasing bending angles can be observed. Below 38°, the geometrical limits were reached, which intrinsically limits the achievable bending angles. For our further investigations, we continued using a 52° angle, as the advantages of slightly more effective bending are outweighed by the possibility of being able to reach higher bending angles. The same experiment was performed for adjusted curing pattern heights as well, and thereby for constant projected areas, and almost identical bending angles were received, compared to the ones depicted in Figure 5a. We do, however, want to mention that the biggest differences between these two variants would be expected for the most gracing incidence angles, which were strongly limited in their bending angles due to inherent, geometrical reasons. Additional studies at beam incidence angles of 52° did, in fact, show a linear increase in the bending effectivity for larger EBC areas for the same overall doses.

Complementary Monte Carlo electron trajectory simulations in Figure 5b illustrate the shift to higher maximum penetration depths with increasing beam incidence angles α, using primary electron energies of 2 keV. This can be nicely explained when having a look at the electron interaction volumes in Figure 5c. In contrast to Figure 2, here, roughly the same volumes are penetrated for different incidence angles. Compared to symmetric 90° incidence angles, the electron interaction volumes for lower α-values are transversely distorted and thereby expand to broader EBC regions and stay within smaller penetration depths. This presumably leads to larger contraction regions on the surface and thereby increases the imbalance leading to stronger controlled bending. In summary, a rather gracing incidence is theoretically favourable, but strongly limits forward bending abilities due to geometrical reasons, making 52° a practicable compromise.

Finally, we want to comment on the observation, that varying PoPs and DTs for the curing procedure at identical overall doses showed only minor deviations. Details on the implications of these parameters can therefore be found in the first section of the supporting information with Figure S1 illustrating the dependency of the bending angles on PoP and DT.

**Figure 6:**
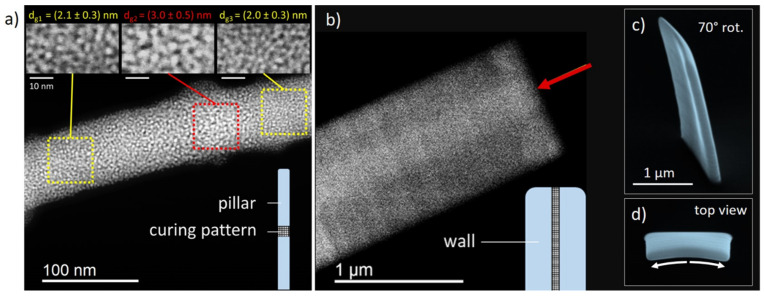
Structural effects by EBC. (**a**) shows a TEM high-angle annular dark field (HAADF) image of a single pillar, which was EBC treated in the centre, as shown in the bottom right scheme. While the varying contrast for the EBC region is immediately evident, the close-ups on top reveal the expected grain growth. (**b**) shows a vertical wall, which was electron beam cured via a rectangular strip from top to bottom (curing schematic included) with the cured area indicated by a red arrow; corresponding SEM images in (**c**) rotated side view and (**d**) top view illustrate the slight folding and tilting induced by the EBC process.

**Structural Implications**—**Transmission Electron Microscopy**—To characterize the inner structure of EBC regions, which were irradiated at 2 keV, transmission electron microscopy (**TEM**) was performed, with a focus on grain sizes and density variations. Figure 6a shows a dark-field image of a single pillar, which was locally modified via EBC with a dose of $8.6\;C/{\mathrm{cm}}^{2}$ (see bottom right scheme). As evident, the cured region reveals brighter contrast and slightly larger platinum grains, compared to uncured regions above/below EBC areas, further confirmed by close-ups of the three indicated regions as insets. Measurements of grain size diameters $d_{g}$ revealed minor growth of the grain size between uncured regions with $d_{g1\;\left(uncured\right)}=\left(2.1\;\pm 0.3\right)\;\mathrm{nm}$ and $d_{g3\;\left(uncured\right)}=\left(2.0\;\pm 0.3\right)\;\mathrm{nm}$ and cured regions with $d_{g2\;\left(cured\right)}=\left(3.0\;\pm 0.5\right)\;\mathrm{nm}$. This is in full agreement with the current understanding of EBC, which first leads to grain growth due to the ongoing dissociation of incompletely fragmented precursor molecules [44]. The second regime, where the carbon matrix is modified [22,36] is not relevant here, as the applied doses are much lower. Figure 6b shows a freestanding wall, which was electron beam cured by using a vertical patterning strip (see bottom scheme) with a dose of 8.3 C/cm², leading to a slightly folded and tilted morphology, as shown by the SEM images in rotated side view (c) and top view (d). The central scanning-TEM-based, high angular-annual-darkfield (STEM-HAADF) image reveals a clear contrast variation between cured (red arrow) and uncured areas, which fully verifies the localized EBC effect.

**Figure 7:**
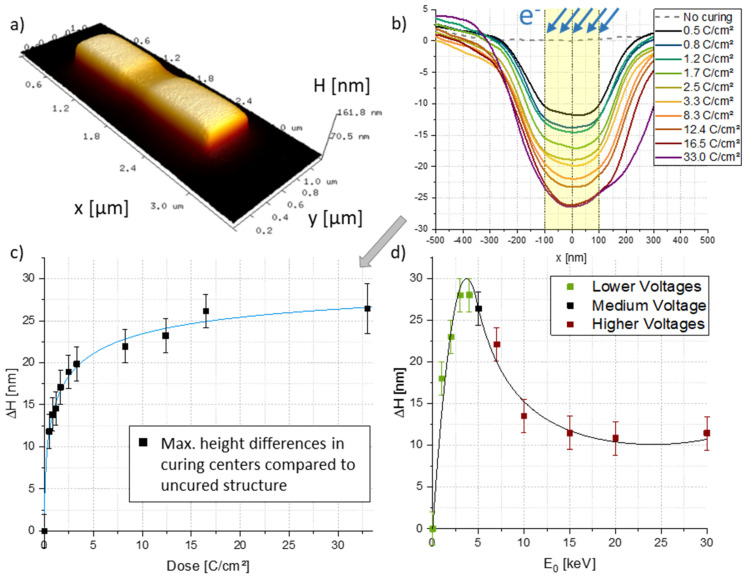
EBC-induced volume losses. (**a**) 3D AFM height image of an exemplary pad after high-dose EBC with a rectangular patterning strip, which immediately reveals the volume loss. (**b**) shows extracted height profiles for different EBC doses, together with the electron beam direction (blue arrows) and the used patterning width of 200 nm (shaded yellow), which is distinctly smaller than the finally arising volume loss area due to complex electron trajectories. (**c**) gives the dose-dependent, maximum height loss, which again reveals a saturation tendency in agreement with bending possibilities and EBC theory. (**d**) summarizes the energy-dependent height loss, which also reveals a very similar behaviour as found for the energy-dependent bending experiments in Figure 2a.

**Volume Losses**—**Atomic Force Microscopy**—After confirming the impact on the nano-granular structures via TEM, we focus on volume loss effects after EBC via atomic force microscopy (**AFM**). We deposited flat, 2000 nm×500 nm rectangular structures, with ≈135 nm heights, on $\mathrm{Si}-{\mathrm{SiO}}_{2}$ substrates. The fundamental idea was for them to mimic horizontal wall structures, to prevent EBC deformations through bending and only observe possible volumetric changes. These pads were then electron beam cured by using 2 keV, identical PoPs/DTs and incidence angles of 52° in relation to the element surface, while varying the doses via the number of passes, to provide comparability for vertical wall studies. A representative 3D AFM image after high-dose EBC is shown in Figure 7a, which immediately confirms the assumed volume loss at the curing site. Extracted height profiles showed the monotonous dependency of the volume loss on EBC doses (Figure 7b), where height decreases of more than 25 nm between as-deposited and cured structures were observed. The figure also shows that for curing strips of 200 nm width, the rectangular base structures were altered over a width of >500 nm due to the extent of the interaction volumes for inclined electron beams, as discussed before. Figure 7c shows the height loss more clearly, which follows an exponential trend that approaches a certain maximum. This is in full agreement with the observed limit for achievable bending angles (compare Figure 4) and with the underlying theory of an ongoing dissociation, which, at some point, has to stop, once fragmentation in the exposed volume is completed. In the following, we studied the height loss for varying $E_{0}$ at a constant dose of 12.4 C/cm², which revealed a clear maximum around 4 keV, as evident in Figure 7d. The overall trend is qualitatively similar to the initial bending experiments, presented in Figure 2a, which suggests that the volume loss itself is strongly involved as originally assumed. However, we want to mention, that the results for higher energies, presented in Figure 7d, have to be taken with care, as volume loss effects in the lower half could lead to a delamination from the substrate, which cannot be accessed via AFM. Finally, we analysed the mean roughness of the surfaces on different areas of partially cured structures. Measurements of the mean surface roughness $R_{a}$ revealed similar values for uncured ($R_{a}=2.6\;\pm \;0.3\;\mathrm{nm}$) and cured areas ($R_{a}=2.3\;\pm \;0.4\;\mathrm{nm}$), while being widely independent of EBC energies and doses. In conclusion, the here confirmed volume loss is qualitatively similar to energy- and dose-dependencies for initial bending experiments and fits well into the general model.

**Figure 8:**
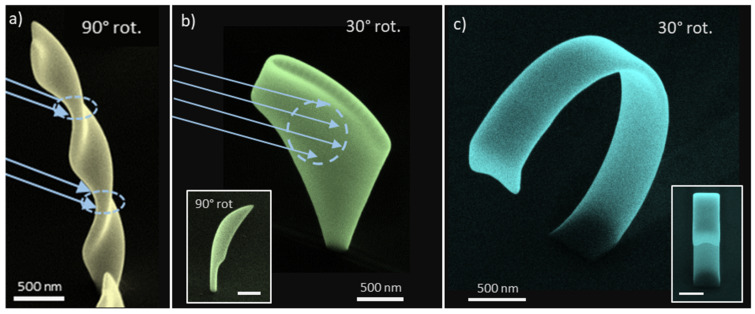
Advanced EBC modified 3D architectures. (**a**) Screw-like geometry with two EBC modifications at indicated regions using rectangular patterning strips. (**b**) Diamond-shaped base structure which was treated with a circular EBC pattern, depicted from a rotated and a side view (bottom left inset). (**c**) shows a base structure, composed of an inclined wall and a vertical top element, further modified by multiple, rectangular EBC strips from top to bottom to generate an overhanging geometry. The inset on the bottom right illustrates the strong downwards bending of the element in a front view under a 52° tilt angle.

**Advanced EBC**—As an expansion to the aforementioned curing procedures, more advanced base elements were deposited via FEBID and exposed to various EBC patterns. Figure 8a shows a screw-like structure with a constant width of W=300 nm and a height of H≈3 µm, that was deposited as a twisted vertical base structure. Two rectangular curing strips at the marked spots created the pictured shape with two visible kinks. This way, an already deposited structure with a comparatively simple geometry could be altered to a desired, more sophisticated shape. In Figure 8b, a sheet-like diamond structure was irradiated within a centrally located circular area, as indicated. A symmetric contraction of the geometry around the curing site was observed, while leaving the angle of the structure foundation unaltered, which can be seen particularly clear in the bottom left inset depicting a side view of the structure. Even though the architectures mentioned in (a) and (b) already constitute important achievements in how localized EBC can be applied, it would theoretically be possible to deposit these structures with direct-write 3D-FEBID alone, although challenging in its reliable calculation and with a strongly increased effort in compiling the geometry construction files. Figure 8c, on the other hand, shows a way how EBC as a post-deposition deformation tool opens up completely new possibilities for FEBID structures. We thereby used a base structure, consisting of an inclined wall and a short vertical element on top, which was then bent against its inclination direction, via multiple rectangular curing strips from top to bottom, until a strongly overhanging geometry was achieved. The originating shape represents an entirely new kind of shape, that cannot be built by 3D-FEBID alone in a vertical, direct-write deposition process.

## 3. Conclusions

We here introduced the post-growth approach of local electron beam curing (EBC) as a morphological tuning tool for the controlled deformation of pre-existing 3D-FEBID objects. This study gives insights into the working mechanism, which is a combination of localized but tuneable volume losses and structural changes in terms of nano-grain growth. Both together are proposed to induce a contraction-based stress situation, which triggers the bending effects. We demonstrated that the extent of the deformations depends on the EBC-induced asymmetry within the structures, which can be tuned by EBC process parameters and patterning strategies. This way, forward and backward bending can be induced, as well as spherical folding and even the fabrication of overhanging structures. For our special case of 100 nm thick ${\mathrm{PtC}}_{x}$ materials, we achieved more than 50° forward and 20° backward bending in a precise and reproducible way. While this study primarily focused on controlled morphological adaption, the structural changes also suggest the possibility of functional tuning. Consequently, this EBC approach has the potential for controlled local adjustment of mechanical, electrical or even thermal properties of low-metal-content 3D-FEBID elements. By that, this study lays the foundation for new options concerning morphological and/or functional post-growth tuning to pave the way for new applications.

## 4. Materials and Methods

**3D Nanofabrication**—FEBID depositions and EBC deformation processes were performed in an SEM/FIB (scanning electron microscope/focused ion beam) dual beam system (Quanta 3D-FEG, FEI, Eindhoven, The Netherlands). The deposition parameters for primary beam energy and beam current were chosen as $E_{0}=5\;\mathrm{keV}$ and $I_{0}=40\;\mathrm{pA}$ at a substrate temperature of $T_{S}\approx \;20\;{}^{\circ}C$, according to pre-tests, to attain uniform growth conditions and to minimize co-deposition^22^. All depositions in this work were fabricated with a platinum precursor (MeCpPt^(IV)^Me_3_, CAS: 94442-22-5) on a 1 cm×1 cm silicon wafer with a 5 nm thick native oxide layer or on a dedicated grid for TEM investigations. The standard FEI gas injection system (**GIS**) was installed at a 52° inclination angle and at a 100 µm distance to the substrate surface, with a projected radial distance of 125 µm to the deposition centre. The gas reservoir was heated to a constant temperature of 45 °C for at least 3 h before each deposition while the precursor flow was opened for over 5 min prior to every fabrication process to reach equilibrium conditions. The chamber pressure thereby changed from $p_{0}\approx 1\times 10^{-6}\;\mathrm{mbar}$ initial chamber pressure to $p_{d}\approx 8\times 10^{-6}\;\mathrm{mbar}\;$ during the process. All 3D-nanoprinted arrays were aligned towards the gas injection system to minimize the influence of the direction of the gas flow^23^, especially concerning the symmetry of vertical walls. Electron beam curing was performed without precursor gas present under a chamber pressure of $p_{E}\approx 1\times 10^{-6}\;\mathrm{mbar}$. Images were taken under a stage tilt-angle of 52°, unless stated differently. Additional analyses were performed via ImageJ^©^, Origin^©^ and Python^©^.

**Monte Carlo Simulations** were performed with Casino^©^ (Version 2.5.1.0).

**Transmission Electron Microscopy**—Transmission electron microscopy (TEM) and scanning transmission electron microscopy (STEM) measurements were performed with a Tecnai F20 (FEI, The Netherlands), operated at 200 keV in monochromated conditions. Analyses were carried out by the software package Digital Micrograph (Gatan Microscopy Suite Version 3.30.2016.0). For TEM studies, the relevant 3D structures were fabricated directly on Cu slit grids (2 mm in diameter), split into half to have access to the central grid bar to prevent any further preparation and transfer steps.

**Atomic Force Microscopy**—Atomic force microscopy (AFM) measurements were performed on a FastScan Bio AFM microscope (Bruker AXS, Auburn, CA, USA) operated by a Nanoscope V controller. As AFM probe an OLTESPA-R3 cantilever (Bruker) with a nominal tip radius of 7 nm and a spring constant of 2 N/m at a resonance frequency of 68 kHz was used in tapping mode in air under soft repulsive conditions. AFM images were acquired with 512×512 pixels, which relates to less than 7 nm digital, lateral resolution. Vertically, the scanner was adjusted to a digital resolution of ≈0.1 nm. AFM data were analysed with NanoScope Analysis 1.80 and Gwyddion (version 2.61), using a 1st order flatten procedure. Roughness data ($R_{a}$) were acquired at an area of 100 nm×300 nm.

## Data Availability

Additional data are presented in the Supplementary Materials and are available on request from the corresponding authors.

## Supplementary Materials

The following supporting information can be downloaded at: https://www.mdpi.com/article/10.3390/nano12234246/s1 and provide additional details on different contents: (1) “PoP and DT Variation” with Figure S1: Impact of point pitch PoP and dwell time DT on EBC efficiency and (2) “Width Variation” with Figure S2: Electron beam cured walls of different widths.
