# Supplementary material for: Controlled Morphological Bending of 3D-FEBID Structures via Electron Beam Curing
Source: Nanomaterials (Basel). 2022 Nov 29;12(23):4246. doi: 10.3390/nano12234246 (PMC9737864; doi:10.3390/nano12234246)
Supplement: Supplementary file 1 [file nanomaterials-12-04246-s001.zip › nanomaterials-2046028-supplementary.pdf]

Supporting Information

Controlled Morphological Bending of 3D-FEBID Structures via Electron Beam Curing

Anna Weitzer [1], Robert Winkler [2], David Kuhness [2], Gerald Kothleitner [1,3] and Harald Plank [1,2,3,*]

[1] Institute of Electron Microscopy and Nanoanalysis, Graz University of Technology, 8010 Graz, Austria
[2] Christian Doppler Laboratory for Direct-Write Fabrication of 3D Nano-Probes, Institute of Electron Microscopy and Nanoanalysis, Graz University of Technology, 8010 Graz, Austria
[3] Graz Centre for Electron Microscopy, Steyrergasse 17, 8010 Graz, Austria
* Correspondence: harald.plank@felmi-zfe.at; Tel.: +43-316-873-8821

Supplement S1: Point Pitch and Dwell Time variation

To test the influence of the step size within the electron beam curing (**EBC**) irradiation grid (point pitch **PoP**) and the dwell time splitting for each patterning pass (dwell time **DT**), an experimental series was conducted. $W \times H \times T = (1000 \times 2000 \times 100)$ nm vertical FEBID walls were used as base elements, which were EBC treated via horizontal patterning strips of $W \times H = (1200 \times 200)$ nm size under a sample tilt angle of 52°. Fig. S1 shows the results for bending angles β in dependency on PoP for two different dwell times $DT = 1$ ms (green) and $DT = 100$ µs (blue). All curing processes were performed at 2 keV primary electron energy, 48 pA primary electron current and irradiation doses of $\approx 8\ C/cm^2$. The average difference in bending angles between $DT = 1$ ms and $DT = 100$ µs is $\Delta\beta \approx 0.1°$ and can therefore be neglected. For further EBC experiments, 1 ms was used as the standard dwell time. For point pitches between 5 nm and 50 nm, a minor increase in bending angles was observed, although with a limited impact of $\Delta\beta \approx 1°$. As the point pitch only seems to play an insignificant role concerning the efficiency of EBC, 5 nm were chosen as the standard parameter as, from an optical point of view, this value provided the smoothest fold with the least indentations at curing sites.

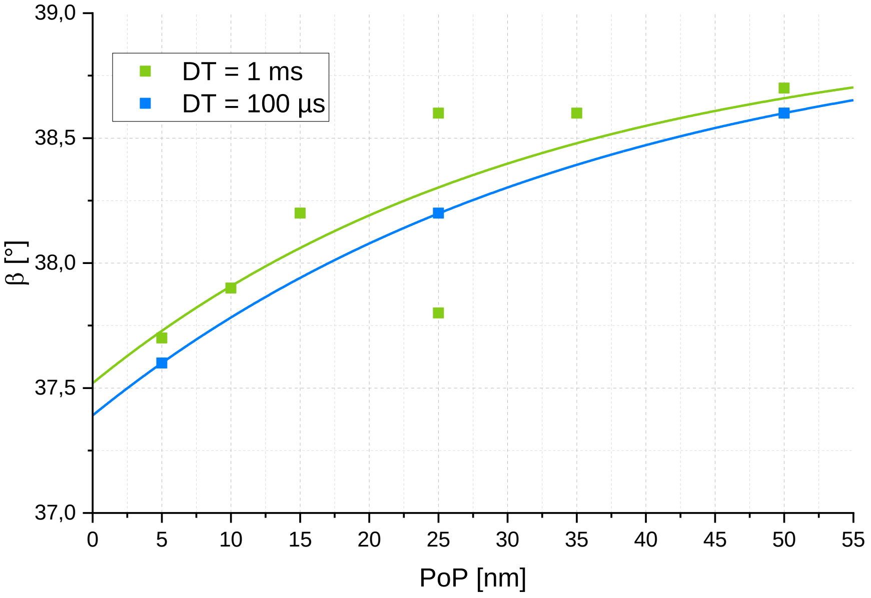

Figure S1: Impact of point pitch PoP and dwell time DT on EBC efficiency. Bending angles β are depicted depending on PoP for two different dwell times of DT = 1 ms (green) and DT = 100 µs (blue).

Supplement S2: Width Variation

To show the applicability of this targeted bending method via EBC to elements of different dimensions, a series of walls with varying widths was deposited via FEBID and electron beam cured at 2 keV and 48 pA with irradiation doses of ≈ $8\ \mathrm{C/cm^2}$ as illustrated in Fig. S2. The bending angles showed deviations of up to 5°, but a significant trend could not yet be discerned from the experimental data. We do, however, expect a more detailed insight into possible differences in the effectivity of the bending behaviour depending on the element width from ongoing investigations.

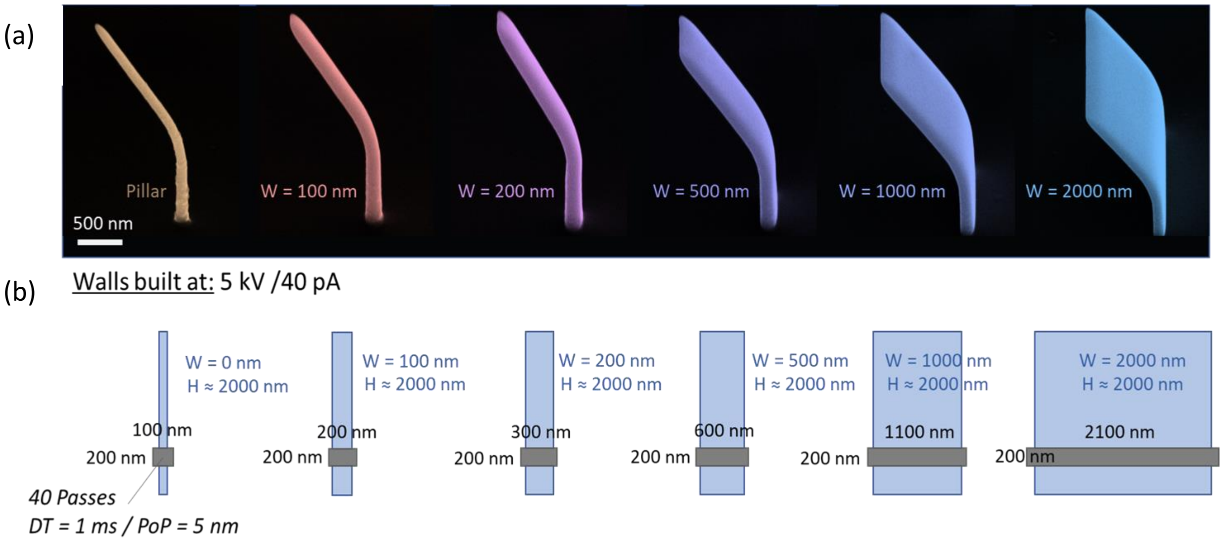

Figure S2: Electron beam cured walls of different widths. (**a**) SEM images of bent walls from point deposition (left) to width W = 2 µm (right) in a side view and (**b**) schematics of the used EBC patterns, with base element walls in blue and electron beam cured areas in grey.
